# Seasonality of allergic diseases: Real‐world evidence from a nationwide population‐based study

**DOI:** 10.1002/iid3.316

**Published:** 2020-06-13

**Authors:** Young Chan Lee, Hyun Jeong Ju, Jin‐woo Kwon, Jung Min Bae

**Affiliations:** ^1^ Department of Otolaryngology‐Head and Neck Surgery, School of Medicine Kyung Hee University Seoul Korea; ^2^ Department of Dermatology, St. Vincent's Hospital, College of Medicine The Catholic University of Korea Seoul Korea; ^3^ Department of Ophthalmology, St. Vincent's Hospital, College of Medicine The Catholic University of Korea Seoul Korea

**Keywords:** allergic conjunctivitis, allergic rhinitis, asthma, atopic dermatitis, atopy, eczema

## Abstract

**Introduction:**

Seasonal variations of allergic diseases have been of great interest in clinical practice, but large‐scale epidemiological data in the real world is lacking.

**Methods:**

We conducted a nationwide, population‐based, cross‐sectional study using the Korean National Health Insurance claims database to examine the seasonalities of allergic rhinitis (AR), asthma, allergic conjunctivitis (AC), and atopic dermatitis (AD). In addition, we investigated the correlations between the monthly patient numbers of each disease and climate factors such as daytime length, temperature, daily temperature range, humidity, solar radiation, rainfall, UVA dose, UVB dose, and PM10.

**Results:**

The highest seasonal variation was identified in AC, followed by AR, asthma, and AD. AR was most prevalent in September and least prevalent in July and was positively correlated with a daily temperature range. Asthma had peaked in the winter and spring and was negatively correlated with both temperature and humidity. AC had dual peaks in May and September and the valley in winter. AD was prevalent between May and August with the lowest visits in winter and positively correlated with temperature.

**Conclusions:**

We demonstrated a clear seasonality of four allergic diseases. Korea is located in a temperate region with four distinct seasons, with 50 million people all having a single health insurance system. Therefore, our data reflects all hospital visits in Korea with the least chance for selection bias.

## Graphical abstract

Seasonal variations of allergic diseases have been of great interest in clinical practice, but there is little large‐scale epidemiological data in the real world. We conducted a nationwide, population‐based, cross‐sectional study using the Korean National Health Insurance claims database to examine the seasonalities of allergic rhinitis, asthma, allergic conjunctivitis, and atopic dermatitis. We revealed a clear seasonality of four allergic diseases and identified associations with various climate factors.

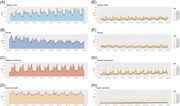

## 1 INTRODUCTION

Allergic diseases are one of the largest medical problems worldwide. Seasonal variations of allergic diseases have been of great interest in clinical practice, but there is little large‐scale epidemiological data. In the present study, we examined the seasonalities of allergic rhinitis (AR), asthma, allergic conjunctivitis (AC), and atopic dermatitis (AD) using the Korean National Health Insurance (NHI) claims database and their association with possible climate factors.

## 2 MATERIALS AND METHODS

We conducted a nationwide, population‐based, cross‐sectional study using entries from the Korean NHI claims database between January 2010 and December 2018. We identified all patients from the database who received a principal medical diagnosis of one of the following diseases: AR (International Classification of Diseases, 10th revision code J30), asthma (J45), AC (H101), or AD (L20). The seasonal ratio was calculated as the ratio of the highest to the lowest number of patients of months each year to compare the degree of seasonality. As each month had a different number of working days regarding national holidays or vacation, we standardized the monthly patient number of each disease by the patient number with diabetes mellitus (E10‐15, and R81) under the assumption that diabetes mellitus would have the least seasonal variation. Subgroup analyses were also conducted by the age group. In addition, we investigated the correlations between monthly patient numbers and climate factors such as daytime length, temperature, daily temperature range, humidity, solar radiation, rainfall, UVA dose, UVB dose, and particular matter 10 (PM10) obtained from the Korea Meteorological Administration. This study was approved by the Institutional Review Board of St Vincent's Hospital (VC19ZESI0179).

## 3 RESULTS

**Figure 1:**
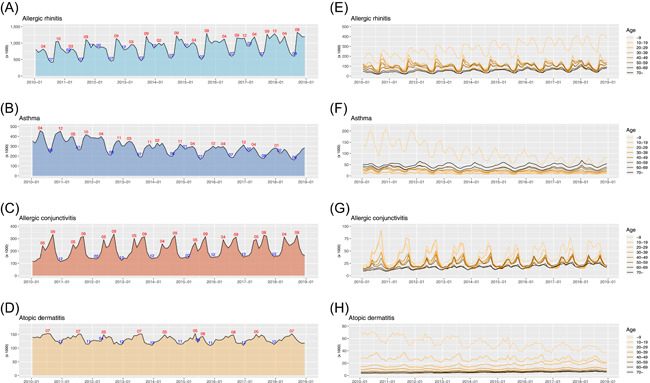
Seasonality of four allergic diseases. A‐D, The standardized monthly patient numbers of four allergic diseases from 2010 to 2018 in Korea. E‐H, Subgroup analyses by age group (0‐9, 10‐19, 20‐29, 30‐39, 40‐49, 50‐59, 60‐69, and 70+ years)

The highest seasonal variation was identified in AC (seasonal ratio: 2.43), followed by AR (2.38), asthma (1.75), and AD (1.33). All of them had their highest prevalence in the 0 to 9 age group, and the highest seasonality was observed in 10 to 19 age group (AC: 3.87, AR: 3.35, asthma: 2.53, and AD: 1.52), which decreased as the age increased (Figure 1).

**Figure 2:**
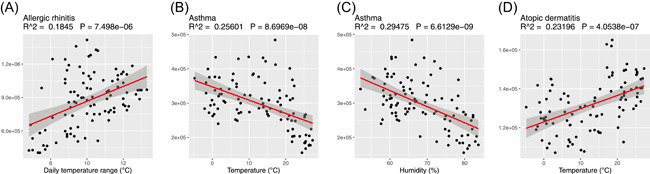
Correlations between monthly patient numbers of various allergic diseases and climate factors. A, Allergic rhinitis and daily temperature range. B, Asthma and temperature. C, Asthma and humidity. D, Atopic dermatitis and temperature

The months with the highest and lowest patient numbers varied among diseases, and several climatic factors were associated with seasonality of disease (Figure 2). AR was most prevalent in September and least prevalent in July and was positively correlated with daily temperature range (*R* ^2^ = .1845; *P* < .001). Asthma had peaked in the winter and spring and valleys in summer and was negatively correlated with both temperature (*R* ^2^ = .26; *P* < .001) and humidity (*R* ^2^ = .29; *P* < .001). AC had dual peaks in May and September and the valley in winter (Figure 1C). AD was prevalent between May and August with lowest visits in winter and positively correlated with temperature (*R* ^2^ = .23; *P* < .001).

## 4 DISCUSSION

Korea has a single health insurance system so our data reflect all hospital visits of 50 million people with the least chance of selection bias. Despite the clear seasonality in all four allergic diseases, differences were evident among them. The seasonality of AC and AR could be explained by the fluctuating temperature between day and night in autumn with a corresponding increase in aeroallergens, such as pollen.^1^, ^2^ On the other hand, exacerbation of asthma could be caused by cold winter temperatures or increased viral infection.^3^ In general, AD is aggravated by low temperature and dry air, but our data indicate that summer‐type AD that worsens in hot and humid weather might have contributed more to the observed seasonality.^4^

The main limitation of our study is that we could not divide the diseases into seasonal and perennial cases. Also, the causality of each climatic factor could not be ascertained. Nevertheless, we revealed a clear seasonality of four allergic diseases and identified associations with various climate factors using a nationwide, population‐based database.

## CONFLICT OF INTERESTS

The authors declare that there are no conflict of interests.

## AUTHOR CONTRIBUTIONS

All authors had full access to all data and accept responsibility for the integrity and accuracy of the data analysis. Study concept and design: JMB and YCL. Acquisition, analysis, and interpretation of the data: HJJ, JK, and JMB. Drafting of the manuscript: HJJ and YCL. Critical revision of the manuscript in terms of important intellectual content: JMB and JK. Statistical analysis: JMB. Administrative, technical, or material support: JMB and JK. Study supervision: JMB and YCL.

## Data Availability

The data that support the findings of this study are available from the corresponding author upon reasonable request.
